# Impact of a Citywide Sanitation Program in Northeast Brazil on Intestinal Parasites Infection in Young Children

**DOI:** 10.1289/ehp.1002058

**Published:** 2010-08-12

**Authors:** Mauricio L. Barreto, Bernd Genser, Agostino Strina, Maria Gloria Teixeira, Ana Marlucia O. Assis, Rita F. Rego, Carlos A. Teles, Matildes S. Prado, Sheila M.A. Matos, Neuza M. Alcântara-Neves, Sandy Cairncross

**Affiliations:** 1 Instituto de Saúde Coletiva; 2 School of Nutrition; 3 Department of Preventive Medicine, Medical School and; 4 Institute of Health Sciences, Federal University of Bahia, Salvador, Brazil; 5 Department of Infectious and Tropical Diseases, London School of Hygiene and Tropical Medicine, London, United Kingdom

**Keywords:** Ascaris, Brazil, developing countries, Giardia, sanitation intervention, sewer, Trichuris, urban health

## Abstract

**Background:**

Sanitation affects health, especially that of young children. Residents of Salvador, in Northeast Brazil, have had a high prevalence of intestinal parasites. A citywide sanitation intervention started in 1996 aimed to raise the level of sewer coverage from 26% to 80% of households.

**Objectives:**

We evaluated the impact of this intervention on the prevalence of *Ascaris lumbricoides, Trichuris trichuria*, and *Giardia duodenalis* infections in preschool children.

**Methods:**

The evaluation was composed of two cross-sectional studies (1998 and 2003–2004), each of a sample of 681 and 976 children 1–4 years of age, respectively. Children were sampled from 24 sentinel areas chosen to represent the range of environmental conditions in the study site. Data were collected using an individual/household questionnaire, and an environmental survey was conducted in each area before and after the intervention to assess basic household and neighborhood sanitation conditions. Stool samples were examined for the presence of intestinal parasites. The effect of the intervention was estimated by hierarchical modeling, fitting a sequence of multivariate regression models.

**Findings:**

The prevalence of *A. lumbricoides* infection was reduced from 24.4% to 12.0%, *T. trichuria* from 18.0% to 5.0%, and *G. duodenalis* from 14.1% to 5.3%. Most of this reduction appeared to be explained by the increased coverage in each neighborhood by the sewage system constructed during the intervention. The key explanatory variable was thus an ecological measure of exposure and not household-based, suggesting that the parasite transmission prevented by the program was mainly in the public (vs. the domestic) domain.

**Conclusion:**

This study, using advanced statistical modeling to control for individual and ecological potential confounders, demonstrates the impact on intestinal parasites of sanitation improvements implemented at the scale of a large population.

The importance of water supply and sanitation in controlling enteric infections, as well as their contribution to poverty eradication, was recognized by the international community when coverage targets for both were included in the Millennium Development Goals (MDG). Indeed, sanitation appears to be no less effective as a public health measure than water supply improvements (Fewtrell et al. 2005), and the promotion of sanitation and hygiene has emerged as one of the most cost-effective possible interventions against high-burden diseases in developing countries (Laxminarayan et al. 2006).

However, although the MDG target for water supply is likely to be met, sanitation is increasingly off track [World Health Organization(WHO)/United Nations Children’s Fund (UNICEF) 2008]. Meanwhile, diarrhea and intestinal parasites continue to exact a heavy toll in developing countries (Hotez et al. 2006; Pruss et al. 2002). However, there is a dearth of rigorous recent evidence for the effectiveness of sanitation programs in preventing disease in large populations. Fewtrell et al. (2005) found only two usable studies for their meta-analysis of sanitation and hygiene programs as a means of reducing diarrhea in developing countries. Most studies in the literature are observational studies (Cairncross and Valdmanis 2006) that may be subject to serious confounding bias (Strina et al. 2003), and the few intervention studies that have evaluated a combined water and sanitation intervention have involved only one or a few small communities (Clasen et al. 2010). We know of no study of the effect of a sanitation intervention on parasites in a large city population of a million people or more. As research on enteric parasite control has become increasingly focused on less-sustainable solutions such as mass chemotherapy (Hotez et al. 2006), such a study is particularly important.

The impacts on diarrhea (Barreto et al. 2007) and intestinal helminths infection in school-age children (Mascarini-Serra et al. 2010) in Salvador, Brazil, from a citywide program to increase access to an adequate sewer system have been presented elsewhere. To summarize, diarrhea prevalence fell by 21% overall and by 42% in the high-risk neighborhoods after the program was implemented. The prevalences of *Ascaris lumbricoides*, *Trichuris trichuria*, and hookworm in schoolchildren were reduced by 25%, 33%, and 82%, respectively. Recent concerns about bias in self-reported incidence of diarrhea (Schmidt and Cairncross 2009) underline the need to confirm such results in studies with harder outcome measures, such as infection with specific pathogens. However, infection with intestinal parasites is far more than a proxy for diarrhea. Awareness is growing of the public health importance and economic consequences of malnutrition in early childhood attributable to poor environmental sanitation (Acharya and Paunio 2008), whether it is occasioned by repeated episodes of diarrhea or by parasite infection. Indeed, *Giardia duodenalis* infection in this setting is associated with diminished growth, even in children suffering no diarrhea at all (Prado et al. 2005). The long-term consequences of parasite infection in one’s early years go beyond diminished growth and include impaired cognitive performance (Berkman et al. 2002).

Large-scale sanitation programs are complex interventions, and their epidemiologic evaluation poses particular challenges. They directly affect the transmission of several diseases in both the public and domestic domains (Cairncross et al. 1996). Substantial externalities apply to this health benefit, insofar as the installation of a toilet benefits not only its users, but also the community at large, by preventing fecal contamination of the collective environment. Sanitation programs also have indirect effects, mediated by ancillary components of the intervention (e.g., paving of streets, improved drainage) and by changes in behavior in response (e.g., improved disposal of children’s stools) (Curtis et al. 1995). Because they take years to implement and cannot normally be randomized, studies of the health effects of sanitation programs can be subject to confounding by a number of covariates, some of which vary with time. Finally, several actors must cooperate for the intervention to bear fruit; at the very least, public investment in sewerage must be matched by the decision by individual households to invest in a toilet and connect it to the network.

We present the results of an epidemiologic study seeking to quantify the impact on infection with *A.lumbricoides, T.trichuria*, and *G.duodenalis* of a sanitation program implemented throughout the city of Salvador in Bahia State, Brazil (population 2.4 million). We evaluated the effect of the intervention using a conceptual framework that guided our approach to analysis, which controls for fixed and time-varying confounders and quantifies the effect of mediating variables (Genser et al. 2006).

## Materials and Methods

### The context

Salvador is in Northeast Brazil, the poorest zone of the country. The city is marked by African influence on its culture and ethnic composition and characterized by inequalities, with great differences in economic, social, and health indicators. In the 1990s, the infant mortality rate was under 30/1,000 live births, with a wide differential between the poorest and the richest areas of the city (Costa et al. 2001).

### Study population

The evaluation was composed of two cross-sectional studies, each of a cohort of children 0–36 months of age at enrollment who were monitored for diarrhea (Barreto et al. 2007). Households were randomly sampled from 24 sentinel areas selected from 111 small areas (consisting of one or more census tracts) using stratified random sampling to represent the poor, mainly unsewered part of the city, which before introduction of the sanitation program in 1997 (the intervention) included about 75% of the population. The sampling and the study design have been described in detail elsewhere (Barreto et al. 1997, 2007; Teixeira et al. 2002). The sentinel areas each comprised a mean of approximately 600 households. A sample of households with children < 3 years of age was selected at random from the full list of households in each sentinel area, and one child in the eligible age range (0–36 months) from each household was randomly selected for enrollment. The preintervention study, carried out before the intervention, began in December 1997 and enrolled 944 children, 681 of whom had one stool sample collected and examined between June and October 1998 (mean age ± SD at the stool collection 25.6 ± 9.6 months). The postintervention study began in October 2003 and enrolled 1,127 children, 976 of whom had a stool sample collected between November 2003 and March 2004 (mean age at the stool collection 20.6 ± 9.8 months). Other characteristics of the two study populations are shown in Table 1. With respect to socioeconomic and environmental variables studied, children omitted from the study populations for lack of a stool sample were not significantly different from children who were included in the analysis.

### The intervention

Before the intervention, approximately 26% of households in the city as a whole were linked to a safe sewer system; others used sanitary alternatives for sewage disposal such as septic tanks, or unsanitary methods such as discharging sewage into the street. In general, sewers served only upper-and middle-class areas in the oldest part of the city. The original objective of the sanitation program, known as Bahia Azul, or Blue Bay, was the control of marine pollution due to the discharge of domestic wastewater. The objective of the program was to increase the population covered with an adequate sewer system to 80%. Roughly half of the total budget of US$440 million, financed mainly by a loan from the Inter-American Development Bank, was used to extend the sewerage network of Salvador, whereas smaller investments were made in water supply improvements, solid waste management, and institutional capacity-building. The construction work was carried out by 140 different construction firms. In Salvador, this involved the laying of more than 2,000 km of sewer pipes, construction of 86 pumping stations, and connection of more than 300,000 households to the sewer network over a period of 8 years (1996–2004). Most of the household connections were made in the later years of the project.

Sanitation projects in developing countries are often linked to hygiene promotion campaigns, which can make it difficult to determine whether impacts on enteric infections are attributable to improved sewer systems or to improved hygiene behaviors. The costs involved in connecting each household to the sewage system were the responsibility of the individual families, and this was a constraint for poor families. To overcome that issue, approximately US$3 million, or roughly 1% of the total budget of the program, was spent on a public education campaign that promoted connections to the sewers and conscientious use of the system, rather than focusing on domestic hygiene promotion. However, the Bahia State Government Audit Commission commented that this campaign “lacked the strength and continuity required to have an impact on connection rates” [Tribunal de Contas do Estado da Bahia (TCEB) 2005]. It can be presumed to have had still less effect on hygiene behavior. Nevertheless, hygiene behavior was studied before and after the intervention and included in the analysis as a possible confounder in case other factors had caused it to change during the intervening years.

### Study variables

#### Outcomes

Stool samples were collected by 15 fieldworkers who knew the households because they were already making twice-weekly home visits. The mother of each child was given a sealed, labeled, and numbered container in which the child’s stool sample was collected on the following day. Those who failed to provide a sample on that day were given two more opportunities to do so. The samples were transported to the laboratory of the Instituto de Saúde Coletiva in an insulated coolbox and examined on the same day. If the sample contained an insufficient quantity of fecal material, the laboratory staff advised the field workers, who collected another sample the following day. A single sample from each child was examined using the Kato–Katz method (Katz et al. 1972) and spontaneous sedimentation. The result was considered positive if eggs (for the helminths) or cysts (for Giardia) were found by either method.

#### Confounding and mediating variables

An individual/household questionnaire was applied at the time of enrollment of each child to assess potential confounding household/child variables. Variables included socioeconomic status, living and sanitation conditions of the household, and child-related variables (birth weight and breast feeding). In addition, anthropometric measurements to assess nutritional status were carried out at baseline, and height-for-age *z*-scores were calculated using the EPINUT program (Epi Info 6.0; Centers for Disease Control and Prevention, Atlanta, GA, USA).

The fieldworkers were trained to check a list of 33 forms of hygienic or unhygienic behavior by the child or the child’s caretaker, if they were observed during the biweekly visits. Based on this information, a composite hygiene behavior score was calculated for each child, and children were classified in a mainly positive group, an intermediate group, and a mainly negative group. Details of the hygiene behavior observation are given elsewhere (Strina et al. 2003).

Missing data, although rare, were dealt with by imputation of the mean for quantitative variables and the mode for categorical ones.

Environmental surveys—using similar methodologies and carried out in 1997 (Milroy et al. 2001) and 2004 in the same sentinel areas and using as their sampling unit the 100-m stretch of road running 50 m to either side of each sampled house—made it possible to build up contextual variables for the sentinel areas. Where necessary, these contextual variables were first converted from numeric (percentage of road tracts in area) to categorical form (1st, 2nd, and 3rd tertile of the percentages). Tertile thresholds were established by combining the two data sets (before and after) so that each area appeared twice. Some of the contextual variables were also used as potentially mediating variables in the present analysis.

### Statistical analysis

**Figure 1:**
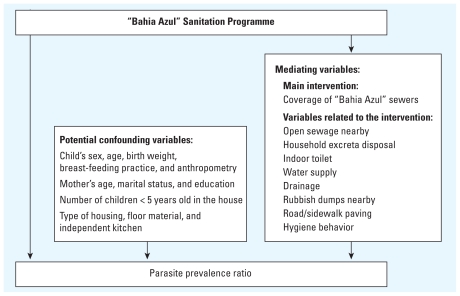
Conceptual model to evaluate the effect of the Bahia Azul sanitation program.

We used a hierarchical modeling strategy according to a conceptual model to evaluate the intervention (Figure 1). The framework assumes that the intervention (Bahia Azul program) mediated its effect on the outcome (parasite prevalence) by changing the distribution of mediating variables such as neighborhood infrastructure (increasing sewerage coverage and improvements in other environmental variables), household living conditions, and hygiene behavior. Furthermore, the model (Figure 1) addresses potential confounding by variables assumed to be independent of the intervention (e.g., age, sex, socioeconomic status). A multivariable mixed-effects Poisson regression model was used to estimate prevalence ratios (PRs) (prevalence after/prevalence before the intervention). Adjustment for child-, household-and sentinel area-specific variables was done by including them as fixed effects in the model together with a gamma-distributed random effect to account for the clustering by sentinel areas.

The evaluation procedure was carried out in several steps. First, we obtained an estimate of the overall effect of the intervention by calculating PRs adjusted for variables that were assumed to be unrelated to the intervention (potential confounders). Estimates of overall effect were also obtained stratified by sentinel area and used to examine effect heterogeneity due to area-specific variables (e.g., baseline prevalence of infection or coverage by the new sewerage system) (Schmid et al. 1991). Second, we implemented a hierarchical effect decomposition strategy to examine by which variables the effect of the intervention had been mediated. We fitted a series of seven models. In addition to the potential confounders, each included one of the following potentially mediating variables (measured separately before and after the intervention and all modeled as categorical variables, except those referring to the sanitary conditions): domestic and peridomestic sanitary conditions, satisfactory drainage, regularity of water supply, absence of rubbish heaps, presence of paved roads or sidewalks, hygienic behavior, and proportion of households in the area connected to the program sewers (Table 1). Only the last of these sought to assess the effect mediated by the direct consequences of the main intervention. The preceding six included potentially mediating variables measuring other changes in environmental infrastructure such as water supply, garbage collection, drainage, household excreta disposal, and changes in hygiene behavior. Finally, we fitted a model which includes all seven of these variables.

For each model we calculated the mediated proportion (MP), that is, the risk reduction explained by the variables in the model according to the formula MP = (PR_unmed_ – PR_med_) ÷ (PR_unmed_ – 1) × 100, where PR_unmed_ and PR_med_ are confounder-adjusted PRs from models without (nonmediated) and with (mediated) the potential mediating variable. The difference of the PR from unity (PR_unmed_ – 1) is a measure of the observed risk reduction; the difference between PRs unadjusted and adjusted for a mediating variable (PR_unmed_ – PR_med_) then represents the part mediated by that variable, and one divided by the other gives the proportion. All statistical analyses were carried out using the statistical software package STATA (version 10.0; StataCorp, College Station, TX, USA).

### Ethics

Ethical approval was granted by the Research Ethics Committee, Instituto de Saúde Coletiva, Universidade Federal da Bahia. The informed consent form was signed by the guardians of all children involved in the study before collection of the stool samples, and free treatment was provided for all children found to be infected.

## Results

**Figure 2:**
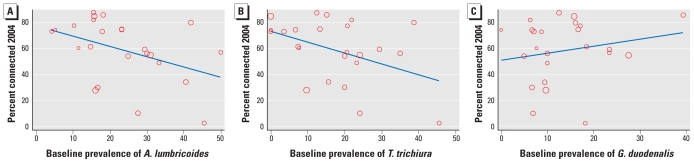
Proportion of households connected to Bahia Azul program sewers in 2004 and baseline (1998) prevalence of (*A*) *A. lumbricoides,* (*B*) *T. trichiura,* and (*C*) *G. duodenalis* by sentinel area; 1,657 children 0–4 years of age, Salvador, Brazil. Each point represents the estimate for a sentinel area, and its size reflects the number of households in that area. The line on each graph is fitted by bivariate linear regression, weighted by the number of households in each sentinel area.

Examining the distribution of mediating variables before and after the intervention (Table 1) showed not only a greatly increased coverage with sewers, but also improved neighborhood environment and infrastructure (e.g., less open sewage, more paved roads, better garbage collection) and improved household conditions (e.g., excreta disposal). The coverage of sewers was not uniform between high- and low-prevalence areas. There was a significant tendency (*p* < 0.05) for lower rates of connection to the sewer system to be achieved in those areas in which the initial prevalence of *A. lumbricoides, T. trichuria* infection was higher, although the same could not be said of *G. duodenalis* infection (Figure 2).

Implementation of the Bahia Azul sewerage program was accompanied by a drop in prevalence from 24.4 to 12.0% for *A. lumbricoides* infection, 18.0 to 5.0% for *T. trichuria* infection, and 14.1 to 5.3% for *G. duodenalis* infection (Table 2). After adjustment for age and sex of the child, PRs were 0.55 [95% confidence interval (CI), 0.43–0.72], 0.35 (95% CI, 0.25–0.49), and 0.42 (95% CI, 0.28–00.65) for *A. lumbricoides, T. trichuria*, and *G. duodenalis* infection, respectively, corresponding to a reduction by roughly half or more than half in the prevalence of infection.

**Figure 3:**
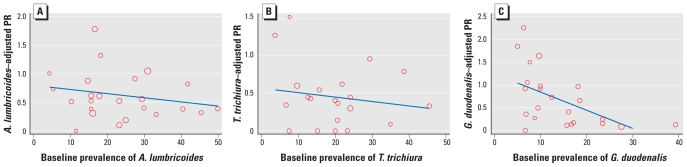
PR of infection with (*A*) *A. lumbricoides,* (*B*) *T. trichiura*, and (*C*) *G. duodenalis* (after vs. before the intervention) plotted against baseline prevalence of the respective parasite in each sentinel area; 1,657 children 0–4 years of age, in Salvador, Brazil, 1997–1998 and 2003–2004. PRs are adjusted for age and sex. Each point represents the estimate for a sentinel area, and its size reflects the number of households in that area. The line on each graph is fitted by bivariate linear regression, weighted by the number of households in each sentinel area.

Analysis stratified by sentinel area showed that the apparent effect of the intervention varied widely among areas; unadjusted PRs ranged from < 0.1 to > 1.0 (data not shown). Figure 3 shows the adjusted PR for each sentinel area plotted against the prevalence of infection in 1998, before the intervention. The plot indicates that the estimated effect of the intervention increased (i.e., PR decreased) with increasing prevalence at baseline for all three species of parasite. For *G. duodenalis* infection, this trend was significant (*p* < 0.01).

Table 3 shows the results of the hierarchical effect decomposition, presenting crude and adjusted PRs (comparing infection after versus before the intervention). Adjustment for a set of confounders besides sex and age (model A) added little to the change in PR already observed on adjustment for age and sex alone (Table 2). Further adjustment for drainage (model B), for solid waste disposal (model D) and for hygiene behavior (model F) made little difference to any of the three parasite PRs and thus did not appear to substantially mediate the effect. By contrast, improvement in domestic and peridomestic sanitary conditions (model G) was associated with reductions in parasite prevalence and could be interpreted as mediating 13.0%, 6.9%, and 16.8% of the overall effect of the intervention on *A. lumbricoides, T. trichuria*, and *G. duodenalis* infection, respectively. The improvement in regularity of water supply (model C) was associated with an MP of the overall effect of 5.4% and 3.5% (for *A. lumbricoides* and *T. trichuria*, respectively), and road and sidewalk paving (model E) appeared to account for 14.6% of the reduction in the prevalence of *A. lumbricoides*. The installation of the program’s sewerage network (model H) appeared to explain 39.7%, 30.3%, and 25% of the reduction in the prevalence of *A. lumbricoides, T. trichuria*, and *G. duodenalis* infection, respectively. The final model (model I), suggested that the observed reduction in prevalence of each parasite after the intervention could be completely explained by the sewerage connections after adjusting for the effect of individual and contextual confounders and mediating variables.

## Discussion

The aim of our study was to evaluate the impact on intestinal parasites in preschool children of the Bahia Azul citywide sanitation program in Salvador. After adjustment for confounders, we found that the implementation of the program was accompanied by a reduction of nearly half (42%) in the prevalence of *A. lumbricoides*, nearly two-thirds (62%) in *T. trichuria* infection, and more than half (59%) in *G. duodenalis* infection in the preschool population of the city, with a tendency for still greater reductions in those areas where the baseline prevalence of infection was highest. This occurred despite the fact that lower sewerage connection rates were achieved in such high-prevalence areas. Multivariate modeling of the change in infection prevalences for the city as a whole showed that the reductions could be fully explained by the program’s interventions and, to a substantial degree, by the increase in coverage of each area with connections to the program’s sewer system. This is the first study that, by using a large set of individual and ecological potential confounders and advanced statistical modeling, demonstrates the impact of improvements in basic sanitation on the intestinal parasites at the scale of an entire city.

No great significance can be attached to the PRs of individual sentinel areas, even where the CIs exclude unity. Cases of infection in a single neighborhood are not statistically independent events, and to draw conclusions from the PR of a single area would be equivalent to a one-to-one comparison (Blum and Feachem 1983). In our analysis of the data set as a whole, we have allowed for clustering by area.

Admittedly, we did not study the same children before and after the intervention, but the differences between the two populations, such as age, were treated as confounders in our models, and we compared the degree of intervention in each area with the changes in prevalence of infection associated with it. The mean elapsed time from enrollment to stool examination, and hence the mean age of the children, was somewhat less for the second study, but this does not explain the reductions in prevalence observed before and after the intervention. The intervention was not randomized, but randomization would not have been politically or ethically acceptable. Rather, the study can be considered as a quasi-experimental health impact evaluation (Briscoe et al. 1986).

The conceptual model was a preliminary effort to deal with the complexity of the intervention. A large range of variables covering different aspects of urban life were defined in advance as confounding or mediating variables (Figure 1). The confounding variables were those that could potentially bias the relationship between the intervention and the outcomes but were not an effect of the intervention. The mediating variables were a series of variables that might be a consequence of the main intervention studied, but may also have changed independently of it. Our study defined individual and contextual variables based on questionnaires, observations (Strina et al. 2003), and environmental surveys (Milroy et al. 2001) carried out using standard protocols by the same staff during the two different periods.

The mediating variables that can be considered as household-based explained only a minority of the heterogeneity of effect of the program: an indoor toilet, absence of open sewage nearby, and an adequate household excreta disposal explained 13% (*A. lumbricoides*), 7% (*T. trichuria*), and 17% (*G. duodenalis*), respectively (Table 3, model G), and hygiene behavior (model F) did not appear to contribute to reductions in infection prevalence at all. Rather, the key explanatory variable—coverage of each sentinel area with connections to the program’s sewers (model H)—was a group-level characteristic of the neighborhood as a whole. This implies that the reduced prevalence of parasites after the program was due to risk factors mainly in the public (as opposed to the domestic) domain (Cairncross et al. 1996).

The overall reduction of *A. lumbricoides* infection by 43% is comparable with the median reductions of 29% found in a previous review (Esrey et al. 1991) of the impact of sanitation and with reductions of 73% and 29% reported by two small intervention studies (Messou et al. 1997; Yang et al. 2000). Our findings are also consistent with a smaller study conducted in the poorest areas of Salvador 20 years ago, which reported 43% and 28% less prevalence of *A. lumbricoides* and *T. trichuria*, respectively, in areas with sewerage and drainage compared with control areas that lacked both (Moraes et al. 2004).

Our finding of a substantial impact on *Giardia* infection (most of which is asymptomatic) assumes added importance in view of the recent finding that even asymptomatic *Giardia* infections contribute to malnutrition (Prado et al. 2005). Little is known about the environmental epidemiology of giardiasis in developing countries, and most of the existing literature is focused on transmission associated with drinking water contamination or the reuse of excreta as fertilizer in agriculture. However, epidemiologic studies of giardiasis in poor urban areas have identified lack of a toilet in the household (Teixeira et al. 2007) and the presence of pools of sewage (Mahfouz et al. 1997) and piles of rubbish near the house (Prado et al. 2003) as important risk factors. The lack of a significant effect of improvements in solid waste and water supply on the prevalence of *G. duodenalis* infection (Table 3, models C and D,) may seem inconsistent with previous studies, but may reflect the modest scale of those improvements in the Bahia Azul program, in contrast with the achievements of the program in sanitation.

The impact of the program is likely to have been more equitable than it might at first appear, as the areas of high baseline risk are also the areas of the city with poorest sanitary conditions (Milroy et al. 2001). Concerns had been voiced locally that the proportion of houses requesting sewer connections was lower, mainly because of financial constraints of the family, in the poorer areas of the city (TCEB 2005). We believe that if a higher level of coverage with sewer connections had been achieved in the high-risk areas, the program would have resulted in an effect greater than what we observed.

Our findings support the importance of sanitation to a number of MDG. In particular, our study suggests that urban sanitation is a highly effective health measure (Waddington et al. 2009). However, there are limits to what can be achieved by individual householders alone, particularly when what is needed are not household toilets (in Salvador, 80% of households already had one) but sewers. Sewer systems require an investment in infrastructure beyond the capabilities of individuals, and it is therefore a public responsibility to ensure that sewer systems are installed. At a typical cost per capita of $160 (WHO/UNICEF 2000), investment in sewerage is too large to be left to cash-strapped municipalities and requires the involvement of central government and its agencies. However, the health sector also has a key role to play, through promotion, advocacy, and regulation, to ensure that toilets and sewers are properly built, used, and maintained, and that their full health benefits are realized by all.

## Figures and Tables

**Table 1:** Distribution of mediating variables evaluated before and after the intervention in the 24 sentinel areas, Salvador, Brazil.

	Percentage of all study children	
	Before the intervention (1997) (*n* = 681)	After the intervention (2003) (*n* = 976)
Variable	Percent (95% CI^a^)	Percent (95% CI^a^)
Indoor toilet—yes	91.8 (89.4–93.7)	98.5 (97.5–99.1)
House excreta disposal—adequate^b^	66.1 (62.4–69.6)	91.2 (89.2–92.9)
Open sewage nearby^c^—no	55.2 (51.4–59.0)	79.6 (76.9–82.1)
Neighborhood with satisfactory drainage system^d^,^e^,^f^		
Third tertile (> 23% of road tracts in area)	26.4 (23.1–29.9)	38.2 (35.2–41.3)
Neighborhood with regular water supply^e^,^f^,^g^		
Third tertile (> 60% of households in area)	21.4 (18.4–24.7)	44.5 (41.3–47.6)
Neighborhood with good garbage collection^e^,^f^,^h^		
Third tertile (> 89% of road tracts in area)	31.6 (28.1–35.2)	35.0 (32.0–38.1)
Neighborhood with paved roads^e^,^f^		
Third tertile (> 84% of road tracts in area)	24.4 (21.2–27.8)	39.7 (36.6–42.8)
Neighborhood with paved sidewalks^e^,^f^		
Third tertile (> 43% of road tracts in area)	25.8 (22.6–29.3)	40.4 (37.3–43.5)
Hygiene behavior^i^ —good	23.8 (20.6–27.2)	30.4 (27.6–33.4)
Connections to Bahia Azul program sewer^e^		
≤ 25% of houses in area	100.0 (99.6–100.0)	7.9 (6.3–9.8)
> 25% to ≤ 50% of houses in area		18.0 (15.7–20.6)
> 50% to ≤ 75% of houses in area		49.8 (46.6–53.0)
> 75% of houses in area		24.3 (21.6–27.1)

a Exact binomial 95% CI.

b Sewer or septic tank.

c Within 30 m of house.

d Rainwater drainage system present and in good maintenance.

e Contextual variables, defined for each sentinel area.

f Tertile values were established by combining the two data sets (before and after) so that each area appeared twice.

g 24-hr water supply.

h No rubbish heaps by the road.

i Children in the group of mainly hygienic behaviors, as observed during home visits (Strina et al. 2003).

**Table 2:** Pre- and postintervention prevalences and crude and adjusted PRs (after versus before the intervention) for *A. lumbricoides, T. trichiura*, and *G. duodenalis*; 1,657 children 0–4 years of age, Salvador, Brazil, 1997–1998 and 2003–2004.

	Prevalence (%)		Unadjusted	Adjusted^a^
Parasite	1998	2003–2004	PR (95% CI^b^)	PR (95% CI^b^)
*A. lumbricoides*	24.4	12.0	0.49 (0.39–0.62)	0.55 (0.43–0.72)
*T. trichiura*	18.0	5.0	0.28 (0.20–0.39)	0.35 (0.25–0.49)
*G. duodenalis*	14.1	5.3	0.38 (0.27–0.53)	0.42 (0.28–0.65)

a Adjusted for age and sex of child.

b Based on analysis by sentinel area.

**Table 3:** PRs of infection with *A. lumbricoides, T. trichiura*, and *G. duodenalis* (after vs. before the intervention) obtained by different regression models: 1,657 children 0–4 years of age, Salvador, Brazil, 1997–1998 and 2003–2004.

	*A. lumbricoides*		*T. trichiura*		*G. duodenalis*	
	PR (95% CI)	MP^a^	PR (95% CI)	MP^a^	PR (95% CI)	MP^a^
PR, unadjusted	0.49 (0.39–0.62)	–	0.28 (0.20–0.39)	–	0.38 (0.27–0.53)	–
Model A: PR adjusted for confounders^b^ and baseline sewerage coverage	0.57 (0.45–0.74)	–	0.38 (0.27–0.53)	–	0.41 (0.27–0.62)	–
Model B: PR adjusted for variables of model A and drainage	0.57 (0.44–0.73)	−1.9	0.34 (0.27–0.48)	−3.7	0.40 (0.27–0.59)	−1.9
Model C: PR adjusted for variables of model A and regularity of water supply	0.60 (0.46–0.79)	5.4	0.40 (0.27–0.59)	3.5	0.41 (0.28–0.61)	−0.3
Model D: PR adjusted for variables of model A and absence of rubbish dumps	0.55 (0.44–0.69)	−5.7	0.35 (0.25–0.47)	−5.3	0.40 (0.27–0.58)	−2.7
Model E: PR adjusted for variables of model A and paved road/sidewalk	0.64 (0.50–0.81)	14.2	0.38 (0.26–0.54)	−0.6	0.39 (0.27–0.57)	−3.1
Model F: PR adjusted for variables of model A and hygiene behavior	0.57 (0.44–0.37)	−1.7	0.38 (0.28–0.52)	−0.2	0.41 (0.27–0.63)	0.3
Model G: PR adjusted for variables of model A and indoor toilet, open sewage nearby and household excreta disposal	0.63 (0.51–0.79)	13.0	0.42 (0.30–0.59)	6.9	0.51 (0.34–0.77)	16.8
Model H: PR adjusted for variables of model A and coverage with program sewerage connections	0.74 (0.34–1.62)	39.7	0.57 (0.41–0.79)	30.3	0.55 (0.26–1.16)	25.0
Model I: PR adjusted for variables of model A and all of the above	1.08 (0.46–2.52)	100.0	1.00 (0.49–2.02)	100.0	0.80 (0.19–3.38)	65.8

a Risk reduction explained by variable(s) in the model MP = (PR_unadj_ – PR_adj_)/(PR_adj_ – 1) × 100.

b Mean age of child during the follow-up, sex, birth weight < 2,500 g, exclusive breast feeding until < 6 months old, height-for-age < −1 *z*-score; age of mother < 20 years at child’s birth, marital status (not married), and education (no schooling or < 4th grade, or 5th–8th grade, versus higher education); number of children < 5 years of age in the house; housing type (shack) and floor (dirt floor), no independent kitchen.
